# Bioactivity of *Carlina acaulis* Essential Oil and Its Main Component towards the Olive Fruit Fly, *Bactrocera* *oleae*: Ingestion Toxicity, Electrophysiological and Behavioral Insights

**DOI:** 10.3390/insects12100880

**Published:** 2021-09-29

**Authors:** Roberto Rizzo, Marco Pistillo, Giacinto Salvatore Germinara, Gabriella Lo Verde, Milko Sinacori, Filippo Maggi, Riccardo Petrelli, Eleonora Spinozzi, Loredana Cappellacci, Valeria Zeni, Angelo Canale, Giovanni Benelli

**Affiliations:** 1 CREA Research Centre for Plant Protection and Certification, SS.113, Km 245,5, 90011 Bagheria, PA, Italy; roberto.rizzo@crea.gov.it; 2 Department of Agricultural Sciences, Food, Natural Resources and Engineering, University of Foggia, Via Napoli 25, 71122 Foggia, Italy; marco.pistillo@unifg.it; 3 Department of Agricultural, Food and Forest Sciences, University of Palermo, Viale delle Scienze, Ed. 5, 90128 Palermo, Italy; milko.sinacori@unipa.it; 4 School of Pharmacy, University of Camerino, Via S. Agostino 1, 62032 Camerino, Italy; filippo.maggi@unicam.it (F.M.); riccardo.petrelli@unicam.it (R.P.); eleonora.spinozzi@unicam.it (E.S.); loredana.cappellacci@unicam.it (L.C.); 5 Department of Agriculture, Food and Environment, University of Pisa, Via del Borghetto 80, 56124 Pisa, Italy; valeria.zeni@phd.unipi.it (V.Z.); angelo.canale@unipi.it (A.C.); giovanni.benelli@unipi.it (G.B.)

**Keywords:** Asteraceae, attract-and-kill, lure-and-kill, carlina oxide, eco-friendly pesticide, green insecticide, protein bait, Tephritidae flies

## Abstract

**Simple Summary:**

In recent years, botanical insecticides based on essential oils (EOs), or their main components, have received much attention as promising tools for Integrated Pest Management, due to their environmental safety and low side effects on non-target organisms. In this study, *Carlina* *acaulis* EO and its main component, carlina oxide, recently proven to be effective larvicidal and adulticidal agents against some insect pests, were analyzed for their toxicity, electroantennographic (EAG), and behavioral responses to adult olive fruit fly (*Bactrocera* *oleae*). The *C*. *acaulis* EO was more toxic to the tephritid than the carlina oxide, and both were more toxic to the same insect pest than EOs from other plant species tested in previous studies. The EAG responses evoked by the EO were significantly higher than those elicited by the carlina oxide. Carlina oxide did not lead to attraction or repellency responses in *B*. *oleae* males and females. Overall, our results highlight the potential employ of *C. acaulis*-borne products in the development of effective eco-friendly “lure and kill” formulations to be used in tephritid management.

**Abstract:**

Among botanical insecticides based on essential oils (EOs) or their main components, *Carlina acaulis* EO and the aromatic polyacetylene carlina oxide, constituting more than 90% of its EO, were recently proven to be effective against the larvae and adults of some insect vectors and pests. In this study, the toxicity of *C. acaulis* EO and carlina oxide were tested on *Bactrocera oleae* adults using a protein bait formulation. The LC_50_ values of the *C. acaulis* EO and carlina oxide were 706 ppm and 1052 ppm, respectively. Electroantennographic (EAG) tests on *B. oleae* adults showed that both carlina EO and oxide elicited EAG dose-dependent responses in male and female antennae. The responses to the EO were significantly higher than those to carlina oxide, indicating that other compounds, despite their lower concentrations, can play a relevant role. Moreover, Y-tube assays carried out to assess the potential attractiveness or repellency of carlina oxide LC_90_ to *B. oleae* adults showed that it was unattractive to both males and females of *B. oleae*, and the time spent by both sexes in either the control or the treatment arm did not differ significantly. Overall, this study points out the potential use of *C. acaulis* EO and carlina oxide for the development of green and effective “lure-and-kill” tools.

## 1. Introduction

*Carlina acaulis* L. (Asteraceae) is a traditional medicinal plant growing in the calcareous mountainous soils of central-southern and eastern Europe and is currently used in herbal products and as a food [1,2]. The main constituent of its essential oil (EO) is the aromatic polyacetylene carlina oxide (>90%) [2,3], also known as 2-(3-phenylprop-1-ynyl)furan. This molecule is synthesized mostly in endodermal secretory ducts occurring in the root cortex [4]. Polyacetylenes deserve particular attention for their promising biological activities [5]. They play a role as phytoalexins, as they contain antifeedant, insecticidal, nematocidal, antimicrobial, and phytotoxic properties [6,7].

Both *C. acaulis* EO and carlina oxide were recently proven to be important larvicidal and adulticidal agents, being highly effective against the filariasis vector, *Culex quinquefasciatus* Say (Diptera: Culicidae), and the housefly, *Musca domestica* L. (Diptera: Muscidae) [8,9]. When encapsulated into nanoformulations, both carlina oxide and *C. acaulis* EO showed acute and/or sublethal toxicity on the larvae of *Lobesia botrana* (Denis & Schiffermüller) (Lepidoptera: Tortricidae) and *C. quinquefasciatus* [10,11]. Once formulated in protein baits, the *C. acaulis* EO was toxic to Mediterranean fruit fly, *Ceratitis capitata* (Wiedemann) (Diptera: Tephritidae) adults, and also affected their aggressive behavior at sublethal concentrations [12].

In this study, we tested *C. acaulis* EO and carlina oxide against another tephritid of high economic interest, the olive fruit fly, *Bactrocera oleae* (Rossi) (Diptera: Tephritidae), the main pest in olive groves worldwide [13]. The damages caused by the olive fruit fly depend on the susceptibility of the cultivar [14,15] and leads to a decrease in yield, the depreciation of table olives, and the deterioration of oil quality [16,17,18]. Despite its high economic importance, limited information is available on the potential use of natural environmentally-friendly active ingredients, such as plant EOs, in the lure-and-kill control method against the olive fruit fly [19,20].

The present contribution analyzes the potential of *C. acaulis* EO and its main constituent, carlina oxide, in the development of effective and eco-friendly lure-and-kill formulations. Ingestion tests were carried out, incorporating both *C. acaulis* products in protein baits, for the evaluation of their toxicity on olive fruit fly adults. Furthermore, to shed light on the potential behavioral effects of *C. acaulis* EO and carlina oxide on olive fruit fly, electroantennographic (EAG) and behavioral tests were respectively performed to evaluate the capability of the male and female antennae to perceive them and to investigate the potential attraction or repellency activity of carlina oxide to *B. oleae* adults.

## 2. Materials and Methods

### 2.1. Essential Oil Extraction and Isolation of Carlina Oxide

A commercial batch of *C. acaulis* dry roots (lot no C-010818091018) was purchased from A. Minardi & Figli S.r.l. (Bagnacavallo, Ravenna, Italy; https://www.minardierbe.it accessed on 26 June 2021) and it was obtained from an Albanian accession of spontaneously growing *C. acaulis* plants harvested in 2018. The roots were finely powdered using a grinder from Albrigi (Stallavena, Verona, Italy, mod. E0585) equipped with a 1.5 mm sieve. The roots (1 kg) were soaked overnight in a 10 L glass flask filled with 7 L of distilled water; afterward, they were subjected to hydrodistillation using a Clevenger-type device; heating was performed using a Falc MA mantle (Falc Instruments, Treviglio, Italy). Distillation was carried out until no more EO condensed in the burette (~8 h). The EO, at the time of collection, had a yellowish color and showed a yield of 0.74% (*w*/*w*) and a density of 1.063 g/mL.

### 2.2. Chemical Analysis of C. acaulis EO and Carlina Oxide Purification

An Agilent 6890 N gas chromatograph equipped with a single quadrupole 5973 N mass spectrometer and an auto-sampler 7863 (Agilent, Wilmington, DE, USA) was used for the characterization of the *C. acaulis* EO. The analysis conditions and the identification of the EO chemical constituents followed those applied in the study recently published by Benelli et al. [13]. Moreover, 1.442 g of the *C. acaulis* EO were purified by silica gel column chromatography (70–230 mesh, 60 Å, Merck) with 100% of *n*-hexane, yielding 1.18 g of pure carlina oxide. The chemical structure of carlina oxide was confirmed by NMR analysis, which was conducted using a Bruker Avance 400 Ultrashield spectrometer. The chemical shifts were reported in δ values (ppm) and the coupling constants (*J*) in hertz. For the analysis, tetramethyl silane (TMS) was used as an internal standard. Concerning the preparation of the NMR sample, 20 mg of carlina oxide were diluted in deuterated chloroform. The NMR spectrum was linear with data reported in the literature [8]. Pure carlina oxide was used for toxicity, electrophysiological and behavioral experiments.

### 2.3. Olive Fruit Fly Rearing

The olive fruit fly adults used in the ingestion toxicity and electrophysiology trials originated from the pupae of *B. oleae* reared from drupes collected in two Sicilian organic olive groves (located in Caccamo and S. Giuseppe Jato, Palermo province, Italy). To obtain a progressive emergence of olive fruit fly adults for the laboratory assays, the pupae were kept in plastic boxes (30 × 30 × 15 cm) in a dark climatic room at 8 °C, and every week, groups of about 2000 pupae each were transferred into plastic cages (30 × 30 × 30 cm) and put in a climatic room at 21 ± 1 °C with a 16:8 (L:D) photoperiod. The olive fruit flies used in the behavioral assays were obtained from pupae collected in a Tuscan olive mill (located in Vicopisano, Pisa province, Italy) during November 2020. Controlled conditions (22 ± 1 °C, 55 ± 5% RH, and natural photoperiod) were allowed for pupae maintenance until the emergence of adults. A dry diet (yeast extract and sucrose mixture, at a ratio of 1:10 *w*/*w*) was used as food for olive fruit fly adults, while water was furnished separately using a cotton wick [21,22].

### 2.4. Ingestion Toxicity Assays

Following Rizzo et al. [20], the bioassays were performed with groups of 10 adults (both sexes, 10–15 days old) casually chosen from the main rearing cages. For each *C. acaulis* EO or carlina oxide concentration, five replicates were performed (with a total of 50 adults). The insects were put into transparent plastic boxes (450 mL), covered with a thin mesh to allow air exchange. The olive fruit flies were nourished using different concentrations of *C. acaulis* EO or carlina oxide mixed with 2 mL of an aqueous emulsion, containing 2% of carboxy-methylcellulose sodium salt (Sigma-Aldrich^®^, St. Louis, MO, USA, medium viscosity), 12.5% of sucrose, and 1% of the protein bait Nu-Bait^®^ (Biogard, Grassobbio, Bergamo, Italy). The mucilage was given inside a bakelite cup (∅ = 30 mm), enclosed with a cotton disk (∅ = 30 mm). For every test, a negative control was used to test the viscous carrier without the EO or the carlina oxide. The following concentrations (ppm) were tested for the EO: 78.13, 156.25, 312.5, 625, 1250, 2500, 5000 and 10,000; while for the carlina oxide the concentrations were 156.25, 312.5, 625, 1250, 2500, 5000 and 10,000 ppm. The dead insects were counted every day, while the last check was performed at 4 days. Controlled laboratory conditions [21 ± 1 °C, 45 ± 10% R.H., 16:8 (L:D)] were used for the experiments.

### 2.5. Electroantennography (EAG)

The antennal sensitivity of 15 to 20 days old male and female *B. oleae* to increasing concentrations of *C. acaulis* EO and carlina oxide was assessed following the method used in previously reported works [19,23,24]. To obtain dose-response curves, hexane solutions from 0.001 to 100 µg/µL of EO and carlina oxide were prepared and kept at −20 °C before the assays. A specimen was inserted in a plastic pipette tip (0.1 mL) with a cut end to allow the protrusion of the head. Two glass capillaries filled with 0.1 M KCl saline solution were used as electrodes. The indifferent electrode was inserted into the head of the insect and the recording electrode was put into contact with the tip of an antenna. AgCl-coated silver wires were used to maintain the electrical continuity between the antennal preparation and an AC/DC UN-6 amplifier in DC mode connected to a PC equipped with the EAG 2.0 program (Syntech Laboratories, Hilversum, The Netherlands).

The stimuli were 10 µL of a hexane solution of *C. acaulis* EO or carlina oxide adsorbed onto a filter paper (Whatman no. 1, Brentford, UK) strip (1 cm^2^) inserted into a Pasteur pipette (15 cm long). A disposable syringe was used to insufflate the stimuli into a stream of charcoal-filtered humidified air (500 mL/min) passing through a stainless-steel delivery tube (∅ = 1 cm), with the outlet placed at approximately 1 cm from the antenna. During 1 s, 2.5 cm^3^ of vapor from an odor cartridge was added.

The control (10 µL of hexane) and standard (10 µL of a 10 µg/µL (*Z*)-3-hexen-1-ol solution) stimuli were applied at the beginning of the experiment and after each group of three test stimuli. The intervals between stimuli were 1 min. The test stimuli were applied in ascending doses on five antennae of each sex from five males and five females.

### 2.6. Behavioral Assays

Although the *C. acaulis* EO can be defined as a monocomponent EO with more than 90% of carlina oxide, we proceeded in further purification (~99%) of the latter compound by column chromatography to exclude the possible interference of minor components, such as benzaldehyde and *ar*-curcumene, in the behavioral assays.

The potential attraction or repellency activity of carlina oxide was evaluated on both sexes of *B. oleae* in Y-tube bioassays, following the method by Canale et al. [25]. The tested dose was equal to the LC_90_ value calculated in ingestion toxicity bioassays. Briefly, the system consisted of a Plexiglas unit (200 × 190 × 15 mm) composed of a central tube (90 mm × 15 mm) and two lateral arms (75 mm × 15 mm). To prevent insects from escaping, a sieve inlay was placed in the lateral arms and an extending glass tube was placed 5.25 cm away from the connection. The top of the unit was covered with a glass panel. Humidified and purified air was provided to the extending glass tube through a Teflon connection at 1 mL/min. The olfactometer was positioned horizontally, at about 80 cm from the ground. Illumination was provided by a vertically hanging cold light lamp (20 W, 250 lux) above (height 60 cm) the olfactometer unit. To start each test, an insect was introduced into the central arm of the Y-tube using a glass vial. The choice for a given cue was recorded if the insect moved to the cue within 3 min of being released and if it engaged in searching behavior on the selected arm for at least 30 s [19,26]. The tested solution was prepared by emulsifying carlina oxide (at the LC_90_ calculated above through ingestion toxicity assays) with DMSO (1:1) and dissolved in deionized water. The formulation was tested vs. a negative control, which consisted of the same solution without carlina oxide. The Y-tube device and illumination conditions used in our experiment were described in detail by Canale et al. [25], with a purified air flux of 1 mL/min. The temperature was 23 ± 1 °C, and the R.H. was 45 ± 5%. To start each test, a fly was introduced into the Y-tube central arm. The potential attractiveness of carlina oxide was evaluated on mated males and females. The formulation was tested at a dosage of 5 μL. The sample was placed on a filter paper dish (∅ = 10 mm, Whatman no. 1). After solvent evaporation (20 s), the cue was moved into a Drechsel bottle (500 mL). A similar filter paper dish treated with the same amount of negative control was introduced into the second Drechsel bottle (500 mL), representing the clean air control. Only first choices of an odor source, where the fly walked into a given arm and remained stationed there at least 30 s, were noted. For each *B. oleae*, the first choice (i.e., the chosen odor source) and the time spent in a given arm were noted. Flies not moving within 3 min of their release were removed and not considered for data analysis [19,26].

At each replicate, the olfactometer arms were flipped around (180°) and the Y-tube was first cleaned with hexane, rinsed with warm water at 35–40 °C, then dipped in a water bath with mild soap for about 5 min, washed with hot water, and finally rinsed with distilled water at room temperature [26]; then the chemicals were renewed. A total of 40 replicates with responsive flies were carried out. For each replicate, each fly was replaced by a new one of the same age. Both sexes were randomly tested every day [27].

### 2.7. Statistical Analysis

In the ingestion toxicity assays, the Abbott’s formula [28] was used to correct the experimental mortality when the control mortality ranged from 1 to 20%; if it was higher, the data were discharged. Next, the LC_10_, LC_30_, LC_50_, and LC_90_ values with an associated 95% confidence interval (CI) and *χ*^2^, were estimated using probit analysis [29].

The amplitude (mV) of the EAG response to each test stimulus was subtracted by the mean EAG response of the two nearest hexane controls to compensate for solvent and/or mechanosensory artifacts [30]. To compensate for the reduction of the antennal responsiveness during the experiment, the resulting EAG response was corrected based on the reduction of the EAG response to the standard stimulus [31]. In the EAG dose-response curves, the activation threshold was the first dose at which the mean EAG response was higher than a “0” value using the Shapiro–Wilk test for normality followed by the one-sample Student’s *t*-test (*p* = 0.05) [32]; the saturation level was assumed to be the lowest dose at which the mean EAG response was equal to or less than the previous one [24]. The male and female EAG responses to each test stimulus were compared using the Student’s *t*-test (*p* = 0.05) for independent samples. In each sex, the mean EAG responses to the same dose of *C. acaulis* EO and carlina oxide were compared using the Student’s *t*-test.

In the behavioral assays, a likelihood *χ*^2^ test with Yates’ correction (*p* = 0.05) was used to compare the proportion of flies choosing carlina oxide or the negative control. For both *B. oleae* males and females, the time spent in the chosen arm was analyzed using the Wilcoxon test (*p* = 0.05), since the data were not normally distributed (Shapiro–Wilk test, *p* < 0.05) nor homoscedastic (Levene’s test, *p* < 0.05). The statistical analyses were carried out with JMP^®^ 13 (SAS), SPSS (version 10.0.7 for Windows, SPSS Inc., Chicago, IL, USA), and Minitab Inc., State College, PA, USA.

## 3. Results

### 3.1. Essential Oil Chemical Analysis

**Figure 1:**
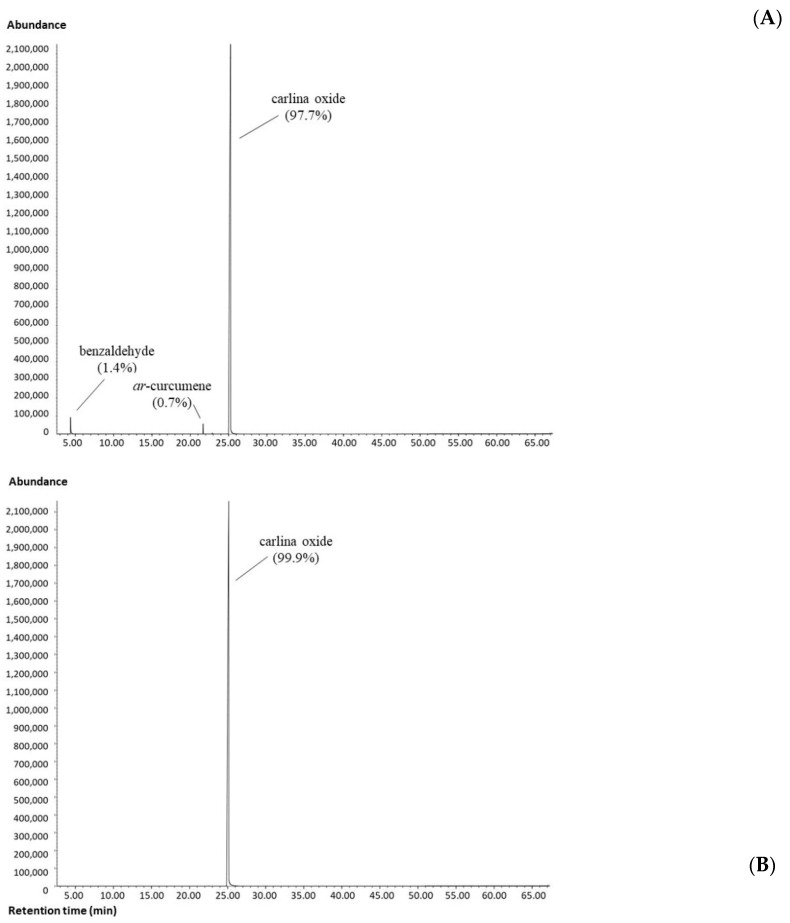
GC-MS chromatograms of *Carlina acaulis* essential oil (**A**) and purified carlina oxide (**B**).

The GC-MS analysis of the *C. acaulis* EO revealed the predominance of carlina oxide (97.7%) in the mixture (Figure 1A), while the remaining identified fraction was composed of the aromatic benzaldehyde (1.4%), the sesquiterpene hydrocarbons *ar*-curcumene (0.7%) and *β*-sesquiphellandrene (0.1%). The total of identified compounds was 99.9%.

This chemical profile overlapped with those of our previous studies [8,9,11,12]. The chromatographic procedure allowed us to increase the purity of the carlina oxide to 99.9% (Figure 1B).

### 3.2. Ingestion Toxicity Bioassays

The probit analysis results showed that the LC_50_ values of the *C. acaulis* EO and carlina oxide were 706.155 and 1052.376 ppm, respectively (Table 1). Comparing the LC values of the EO and carlina oxide, the latter showed a lower toxicity than the EO (Table 1). A significant effect of the kind of tested botanical product (*F_1,64_* = 9.54, *p* = 0.003) and of its concentration (*F_14,64_* = 50.09, *p* < 0.001) was observed.

### 3.3. EAG Experiments

**Figure 2:**
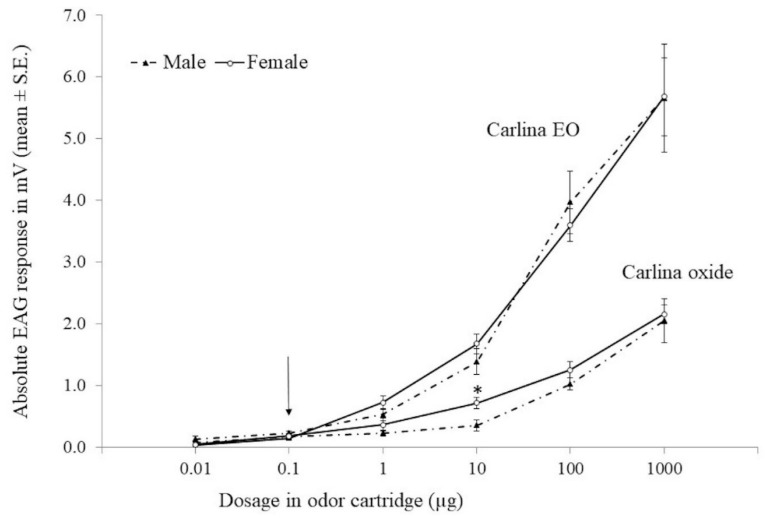
Electroantennogram dose-response curves of *Bactrocera oleae* males and females (*n* = 5) to *Carlina acaulis* essential oil (EO) and its main component, carlina oxide. The arrow indicates the activation thresholds. The asterisk indicates significant differences between male and female EAG responses to carlina oxide at *p* = 0.05 (*t*-test for independent samples).

The EAG responses of *B. oleae* males and females to increasing concentrations of *C. acaulis* EO and carlina oxide are listed in Figure 2. Both stimuli elicited EAG dose-dependent responses in male and female antennae. In both sexes, the activation threshold was 0.1 µg in response to the EO (male *t* = 9.804, *d.f.* = 4, *p* = 0.001; female *t* = 4.103, *d.f.* = 4, *p* = 0.007) and carlina oxide (male *t* = 4.884, *d.f.* = 4, *p* = 0.008; female *t* = 5.074, *d.f.* = 4 *p* = 0.015). The mean male and female EAG responses to the 1000 µg dose of the EO and carlina oxide was higher than those to the 100 µg dose, indicating that saturation of the receptors did not occur at the lowest dose. Except for the 10 µg dose of carlina oxide, which elicited a significantly higher (*t* = 2.773, *d.f.* = 8, *p* = 0.024) mean EAG response in the females than in males, no significant differences were found between male and female EAG responses to the remaining carlina oxide doses (*t* = 0.155–1.342, *d.f.* = 8, *p* = 0.216–0.881) and to all the EO doses (*t* = 0.017–2.003, *d.f.* = 8, *p* = 0.086–0.987) tested. The EAG response to the EO was significantly higher than that to carlina oxide at doses from 1 to 1000 µg (*t* = 2.856–5.705; *d.f.* = 8; *p* = 0.021–0.001) in males, and at doses from 10 to 1000 µg (*t* = 5.109–7.813; *d.f.* = 8; *p* = 0.001) in females.

### 3.4. Behavioral Assays

The Y-tube experiments showed that carlina oxide was unattractive to both male and female *B. oleae*. For both sexes, 21 flies selected the control and 19 the treated arm (male: *χ*^2^ = 0.1, *d.f.* = 1, *p* = 0.718; female: *χ*^2^ = 0.1, *d.f.* = 1, *p* = 0.718).

**Figure 3:**
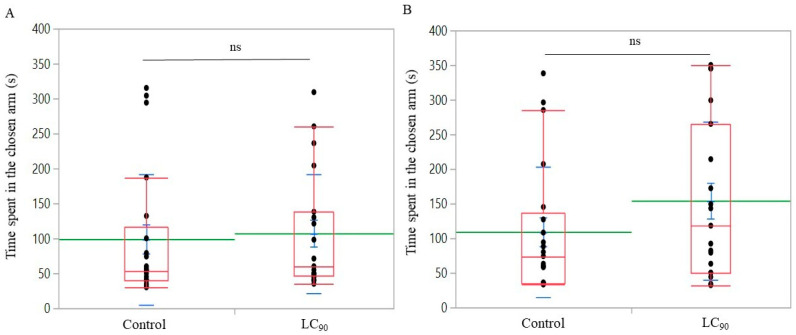
Time spent in the chosen arm by (**A**) males and (**B**) females of *Bactrocera oleae* in Y-tube experiments assessing the tephritid behavioral responses to carlina oxide formulated at its ingestion LC_90_. Red boxplots indicate the median (solid line) within each box and the range of dispersion (lower and upper quartiles and outliers). Means and standard errors are represented by green lines and blue T-bars, respectively. Above the boxplots, ns indicates no significant difference with the control (Wilcoxon test, *p* > 0.05).

The time spent by both sexes in the control or the treatment arm did not differ significantly (male: *χ*^2^ = 0.309, *d.f.* = 1, *p* = 0.578; female: *χ*^2^ = 1.799, *d.f.* = 1, *p* = 0.180) (Figure 3).

## 4. Discussion

Assessing the efficacy of eco-friendly molecules in lure-and-kill programs for establishing effective control strategies against tephritids represents an important challenge [33]. In this framework, the insecticidal efficacy of different EOs incorporated in protein baits against *B. oleae* was highlighted by Canale et al. [22], focusing on the toxicity of *Hyptis suaveolens* (L.) Poiteau, *Rosmarinus officinalis* L. and *Lavandula angustifolia* Miller EOs, as well as by Rizzo et al. [20] for *Pimpinella anisum* L., *Ocimum gratissimum* L., *Thymbra spicata* L. and *Trachyspermum ammi* (L.) Sprague EOs. The present study showed that *C. acaulis* EO and carlina oxide are highly toxic to both sexes of the olive fruit fly when incorporated in protein baits, with both products achieving a significant concentration-dependent effect. The polyacetylenes class encloses a variety of compounds, which showed several biological properties that could be linked to their high reactivity and instability; this was mainly caused by the presence of conjugated C–C triple bonds. Specifically, these compounds undergo fast oxidation, particularly after UV light exposure, and they are highly susceptible to the pH of the medium [7]. There are also studies reporting that polyacetylenes can be activated by sunlight wavelengths less than 400 nm, with an improvement of their toxicity [34]. These compounds are considered photosensitizers, leading to the photodynamic disruption of membranes [35]. Even if the mechanism of action of carlina oxide is not clear, its bioactivity seems to be linked to the C–C triple bond that leads to radical production after UV exposure [36]. Another possible mechanism of action, reported in a previous study, is the alkylation of enzymes [37].

However, based on the LC values, carlina oxide was less toxic to the tephritid than the *C. acaulis* EO (Table 1). These differences may be explained by the presence of minor components in the EO. Therefore, although the bioactivity of EO is related to its major constituent carlina oxide, the minor components seem to play a role in increasing EO toxicity. Moreover, the LC_50_ calculated for *C. acaulis* EO and carlina oxide (706.15 ppm and 1052.37 ppm, respectively) was lower than the LC_50_ calculated for many other EOs, such as *H. suaveolens* (4922 ppm), *R. officinalis* (5107 ppm), *L. angustifolia* (6272 ppm) and *Th. spicata* (2509 ppm). In addition, the LC_50_ calculated for carlina oxide was comparable to that of *O. gratissimum* EO (925 ppm), while the *C. acaulis* EO had an LC_50_ comparable to that of *P. anisum* (771 ppm) and *T. ammi* (633 ppm) [20,22]. Interestingly, the LC_50_ of *C. acaulis* EO reported in the present research for *B. oleae* was lower than the LC_50_ calculated for *C. capitata* in a previous study (1094 ppm, Benelli et al. [12]).

The EAG experiments demonstrated *C. acaulis* EO and carlina oxide’s ability to stimulate the peripheral olfactory system of *B. oleae* adults in a dose-dependent manner. The EAG results showed that in the dose range of 0.01 to 1000 µg, dose-dependent EAG responses, similar in males and females, were elicited by EO (0.03–5.67 mV) and carlina oxide (0.07–2.11 mV), indicating their strong antennal sensitivity to both stimuli. Furthermore, the male and female sensitivities to these stimuli were similar, since no significant differences were found between the EAG responses of both sexes to most of the doses tested. In both sexes, the EAG responses produced by the EO were significantly higher than those elicited by its main component, carlina oxide. Since an electroantennogram is the summation of receptor potentials evoked by an olfactory stimulus from various sensilla on the antennae [38], these differences may be explained by the presence of EAG-active minor components in the EO working independently on separate receptor sites of the antennae. In our study, 99.9% of compounds was identified: 97.7% was carlina oxide, whereas the remaining 2.2% was represented by additional minor components of *C. acaulis* EO, such as benzaldehyde (1.4%), *ar*-curcumene (0.7%) and *β*-sesquiphellandrene (0.1%) (Figure 1A). Benzaldehyde was also identified in the extracts of some olive cultivars that were found to elicit electrophysiological responses in male and female antennae of *B. oleae* [39]. Moreover, the electrophysiological and behavioral activity of plant-volatile terpenes towards tephritid flies, including *B. oleae*, has been demonstrated [40].

Despite their marked electrophysiological activity, in the dose range tested the EO and carlina oxide had a neutral effect on the fly behavior, since no preferential orientation was elicited in the olfactometer bioassays. Relying on behaviorally inactive molecules as active insecticidal ingredients for lure-and-kill approaches can be regarded as a useful advantage, since they will not deter insects from feeding on the protein bait.

Finally, concerning its safety for mammals, recent studies pointed out that the *C. acaulis* EO is cytotoxic to fibroblasts and keratinocytes and mildly toxic to rats [11].

## 5. Conclusions

This study analyzed the potential of *C. acaulis*-borne products as effective ingredients for the development of lure-and-kill tools to manage *B. oleae*. Our results highlighted the adulticidal activity of *C. acaulis* EO and its main component, carlina oxide, at relatively low concentrations. This work also underlined how the LC_90_ of carlina oxide does not lead to behavioral attraction or repellency responses in this key olive pest, despite the clear EAG responses recorded for both sexes. Due to the limited presence of *C. acaulis* in natural habitats, the industrial exploitation of its EO can be warranted by the production of the raw material through cultivation, even if the percentage of carlina oxide in the EO can be affected by cultural practices, such as substrate (hydroponics or field conditions) or fertilization [41,42]. Further studies on the evaluation of mammal safety and potential sub-lethal effects on insects of *C. acaulis* EO and carlina oxide are ongoing [43], as well as insights on the insecticidal activity of nanoformulations in which *C. acaulis* products are encapsulated to preserve their effectiveness over time [10,44] and their field validation.

## Figures and Tables

**Table 1:** Lethal concentrations of the *Carlina acaulis* essential oil (EO) and its major constituent, carlina oxide, formulated in protein baits against adults of the olive fruit fly, *Bactrocera oleae*.

Tested Product	LC_10_ (95%CI) (ppm)	LC_30_ (95%CI) (ppm)	LC_50_ (95% CI) (ppm)	LC_90_ (95% CI) (ppm)	Intercept ± SE	Slope ± SE	Goodness of Fit *χ*^2^ (*d.f.*)
*C. acaulis* EO	190.823 (96.319–288.602)	413.400 (268.900–547.482)	706.155 (530.213–880.887)	2613.202 (1991.810–3939.835)	2.595 ± 0.318	2.255 ± 0.309	12.838 (7) *p* = 0.076 *n.s.*
Carlina oxide	352.606 (240.896–463.244)	672.744 (521.039–821.618)	1052.376 (865.805–1254.674)	3140.863 (2530.428–4178.891)	2.638 ± 0.259	2.698 ± 0.271	7.446 (6) *p* = 0.282 *n.s.*

LC = lethal concentration killing 10% (LC_10_), 30% (LC_30_), 50% (LC_50_), or 90% (LC_90_) of the exposed population. 95% CI = 95% confidence interval. *n.s.* = not significant (*p* > 0.05).

## References

[1] Stojanović-Radić Z., Čomić L., Radulović N., Blagojević P., Mihajilov-Krstev T., Rajković J. (2012). Commercial Carlinae radix herbal drug: Botanical identity, chemical composition and antimicrobial properties. Pharm. Biol..

[2] Strzemski M., Wójciak-Kosior M., Sowa I., Załuski D., Verpoorte R. (2019). Historical and traditional medical applications of *Carlina acaulis* L.-A critical ethnopharmacological review. J. Ethnopharmacol..

[3] Wnorowski A., Wnorowska S., Wojas-Krawczyk K., Grenda A., Staniak M., Michalak A., Wo’zniak S., Matosiuk D., Biała G., Wójciak M. (2020). Toxicity of Carlina Oxide—A Natural Polyacetylene from the *Carlina acaulis* Roots—In Vitro and in Vivo Study. Toxins.

[4] Fritz E., Saukel J. (2011). Anatomy of subterranean organs of medicinally used Cardueae and related species and its value for discrimination. Sci. Pharm..

[5] Negri R. (2015). Polyacetylenes from terrestrial plants and fungi: Recent phytochemical and biological advances. Fitoterapia.

[6] Wat C.K., Prasad S.K., Graham E.A., Partington S., Arnason T., Towers G.H.N., Lam J. (1981). Photosensitization of invertebrates by natural polyacetylenes. Biochem. Syst. Ecol..

[7] Minto R.E., Blacklock B.J. (2008). Biosynthesis and function of polyacetylenes and allied natural products. Prog. Lipid. Res..

[8] Benelli G., Pavela R., Petrelli R., Nzekoue F.K., Cappellacci L., Lupidi G., Quassinti L., Bramucci M., Sut S., Dall’Acqua S. (2019). Carlina oxide from *Carlina acaulis* root essential oil acts as a potent mosquito larvicide. Ind. Crop. Prod..

[9] Pavela R., Maggi F., Petrelli R., Cappellacci L., Buccioni M., Palmieri A., Canale A., Benelli G. (2020). Outstanding insecticidal activity and sublethal effects of *Carlina acaulis* root essential oil on the housefly, *Musca domestica*, with insights on its toxicity on human cells. Food Chem. Toxicol..

[10] Benelli G., Pavoni L., Zeni V., Ricciardi R., Cosci F., Cacopardo G., Gendusa S., Spinozzi E., Petrelli R., Cappellacci L. (2020). Developing a Highly Stable *Carlina acaulis* Essential Oil Nanoemulsion for Managing *Lobesia botrana*. Nanomaterials.

[11] Pavela R., Pavoni L., Bonacucina G., Cespi M., Cappellacci L., Petrelli R., Spinozzi E., Aguzzi C., Zeppa L., Ubaldi M. (2021). Encapsulation of *Carlina acaulis* essential oil and carlina oxide to develop long-lasting mosquito larvicides: Microemulsions versus nanoemulsions. J. Pest Sci..

[12] Benelli G., Rizzo R., Zeni V., Govigli A., Samková A., Sinacori M., Lo Verde G., Pavela R., Cappellacci L., Petrelli R. (2021). *Carlina acaulis* and *Trachyspermum ammi* essential oils formulated in protein baits are highly toxic and reduce aggressiveness in the medfly, *Ceratitis capitata*. Ind. Crops Prod..

[13] Daane K.M., Johnson M.W. (2010). Olive fruit fly: Managing an ancient pest in modern times. Annu. Rev. Entomol..

[14] Malheiro R., Casal S., Cunha S.C., Baptista P., Pereira J.A. (2015). Olive volatiles from Portuguese cultivars Cobrancosa, Madural and Verdeal Transmontana: Role in oviposition preference of *Bactrocera oleae* (Rossi) (Diptera: Tephritidae). PLoS ONE.

[15] Rizzo R., Caleca V., Lombardo A. (2012). Relation of fruit color, elongation, hardness, and volume to the infestation of olive cultivars by the olive fruit fly, *Bactrocera oleae*. Entomol. Exp. Appl..

[16] Tzanakakis M.E. (2006). Insects and Mites Feeding on Olive: Distribution, Importance, Habits, Seasonal Development and Dormancy.

[17] Gucci R., Caruso G., Canale A., Loni A., Raspi A., Urbani S., Taticchi A., Esposto S., Servili M. (2012). Qualitative changes of olive oils obtained from fruits damaged by *Bactrocera oleae* (Rossi). HortScience.

[18] Caleca V., Antista G., Campisi G., Caruso T., Lo Verde G., Maltese M., Rizzo R., Planeta D. (2017). High quality extra virgin olive oil from olives attacked by the olive fruit fly, *Bactrocera oleae* (Rossi) (Diptera Tephritidae): Which is the tolerable limit? Data from experimental ‘Nocellara del Belice’ and ‘Cerasuola’ olive groves in Sicily. Chem. Eng. Trans..

[19] Canale A., Germinara S.G., Carpita A., Benelli G., Bonsignori G., Stefanini C., Raspi A., Rotundo G. (2013). Behavioural and electrophysiological responses of the olive fruit fly, *Bactrocera oleae* (Rossi) (Diptera: Tephritidae), to male- and female-borne sex attractants. Chemoecology.

[20] Rizzo R., Lo Verde G., Sinacori M., Maggi F., Cappellacci L., Petrelli R., Vittori S., Reza Morshedloo M., Yvette Fofiee N.G.B., Benelli G. (2020). Developing green insecticides to manage olive fruit flies? Ingestion toxicity of four essential oils in protein baits on *Bactrocera oleae*. Ind. Crops Prod..

[21] Canale A., Benelli G. (2012). Impact of mass-rearing on the host seeking behaviour and parasitism by the fruit fly parasitoid *Psyttalia concolor* (Szépligeti) (Hymenoptera: Braconidae). J. Pest Sci..

[22] Canale A., Benelli G., Conti B., Lenzi G., Flamini G., Francini A., Cioni P.L. (2013). Ingestion toxicity of three Lamiaceae essential oils incorporated in protein baits against the olive fruit fly, *Bactrocera oleae* (Rossi) (Diptera: Tephritidae). Nat. Prod. Res..

[23] Rotundo G.R., Germinara S., De Cristofaro A., Rama F. (2001). Identificazione di composti volatili in estratti da diverse cultivar di *Olea europaea* L. biologicamente attivi su *Bactrocera olae* (Gmelin) (Diptera: Tephritidae). Boll. Lab. Entomol. Agrar. Filippo Silvestri.

[24] Germinara G.S., De Cristofaro A., Rotundo G. (2009). Antennal olfactory responses to individual cereal volatiles in *Theocolax elegans* (Westwood) (Hymenoptera: Pteromalidae). J. Stored Prod. Res..

[25] Canale A., Benelli G., Germinara G.S., Fusini G., Romano D., Rapalini F., Desneux N., Rotundo G., Raspi A., Carpita A. (2015). Behavioural and electrophysiological responses to overlooked female pheromone components in the olive fruit fly, *Bactrocera oleae* (Diptera: Tephritidae). Chemoecology.

[26] Carpita A., Canale A., Raffaelli A., Saba A., Benelli G., Raspi A. (2012). (Z)-9-tricosene identified in rectal gland extracts of *Bactrocera oleae* males: First evidence of a male-produced female attractant in olive fruit fly. Naturwissenschaften.

[27] Ngumbi E., Jordan M., Fadamiro H. (2012). Comparison of associative learning of host-related plant volatiles in two parasitoids with different degrees of host specificity, *Cotesia marginiventris* and *Microplitis croceipes*. Chemoecology.

[28] Abbott W.S. (1925). A method of computing the effectiveness of an insecticide. J. Econ. Entomol..

[29] Finney D.J. (1978). Statistical Method in Biological Assay.

[30] Raguso R.A., Light D.M. (1998). Electroantennogram responses of male *Sphinx perelegans* hawkmoths to floral and “green-leaf volatiles”. Entomol. Exp. Appl..

[31] Den Otter C.J., Tchicaya T., Schutte A.M. (1991). Effects of age, sex and hunger on the antennal olfactory sensitivity of tsetse flies. Physiol. Entomol..

[32] Germinara G.S., Pistillo M., Griffo R., Garonna A.P., Di Palma A. (2019). Electroantennographic Responses of *Aromia bungii* (Faldermann, 1835) (Coleoptera, Cerambycidae) to a Range of Volatile Compounds. Insects.

[33] Scolari F., Valerio F., Benelli G., Papadopoulos N.T., Vaníčková L. (2021). Tephritid fruit fly semiochemicals: Current knowledge and future perspectives. Insects.

[34] Arnason T., Swain T., Wat C.K., Graham E.A., Partington S., Towers G.H.N., Lam J. (1981). Mosquito larvicidal activity of polyacetylenes from species in the Asteraceae. Biochem. Syst. Ecol..

[35] Waksmundzka-Hajnos M., Sherma J., Kowalska T. (2008). Thin Layer Chromatography in Phytochemistry.

[36] Wink M. (2012). Medicinal plants: A source of anti-parasitic secondary metabolites. Molecules.

[37] Herrmann F., Hamoud R., Sporer F., Tahrani A., Wink M. (2011). Carlina oxide—A natural polyacetylene from *Carlina acaulis* (Asteraceae) with potent antitrypanosomal and antimicrobial properties. Planta Med..

[38] Schneider D. (1962). Electrophysiological investigations on the olfactory specificity of sexual attracting substances in different species of moth. J. Insect Physiol..

[39] Malheiro R., Ortiz A., Casal S., Baptista P., Pereira J.A. (2015). Electrophysiological response of *Bactrocera oleae* (Rossi) (Diptera: Tephritidae) adults to olive leaves essential oils from different cultivars and olive tree volatiles. Ind. Crops Prod..

[40] Anfora G., Vitagliano S., Germinara G.S., Latella C., Mazzoni V., Rotundo G., De Cristofaro A. (2012). Electrophysiological and behavioural activity of plant volatile terpenes in three tephritid flies. IOBC/WPRS Bull..

[41] Strzemski M., Dresler S., Sowa I., Czubacka A., Agacka-Mołdoch M., Płachno B.J., Granica S., Feldo M., Wójciak-Kosior M. (2020). The impact of different cultivation systems on the content of selected secondary metabolites and antioxidant activity of *Carlina acaulis* plant material. Molecules.

[42] Strzemski M., Dzida K., Dresler S., Sowa I., Kurzepa J., Szymczak G., Wójciak M. (2021). Nitrogen fertilisation decreases the yield of bioactive compounds in *Carlina acaulis* L. grown in the field. Ind. Crops Prod..

[43] Benelli G., Ceccarelli C., Zeni V., Rizzo R., Lo Verde G., Sinacori M., Boukouvala M.C., Kavalieratos N.G., Ubaldi M., Tomassoni D. (2022). Lethal and behavioural effects of a green insecticide against an invasive polyphagous fruit fly pest and its safety to mammals. Chemosphere.

[44] Pavoni L., Pavela R., Cespi M., Bonacucina G., Maggi F., Zeni V., Canale A., Lucchi A., Bruschi F., Benelli G. (2019). Green micro-and nanoemulsions for managing parasites, vectors and pests. Nanomaterials.

